# LRRC8D Suppresses Prostate Cancer Growth and Enhances Platinum Sensitivity via Modulation of CAV-1/STAT3 Signaling

**DOI:** 10.3390/membranes16060198

**Published:** 2026-06-08

**Authors:** Rong Xu, Xue Shui, Hao Han, Yanzi Xing, Caiqin Zhang, Pengpeng Wu, Yong Zhao, Dengxu Tan, Jing Qin, Xiaoming Wang, Changhong Shi

**Affiliations:** 1 Division of Cancer Biology, Laboratory Animal Center, The Air Force Military Medical University, Xi’an 710032, China; xurongkeke@126.com (R.X.); pamelashui@foxmail.com (X.S.); hanhao95@126.com (H.H.); 18791686309@163.com (Y.X.); zhangcaiqin@fmmu.edu.cn (C.Z.); wpp217@163.com (P.W.); zhaoyong@fmmu.edu.cn (Y.Z.); tandengxu@fmmu.edu.cn (D.T.); qinjingjd@fmmu.edu.cn (J.Q.); 2 Department of Geriatrics, Xijing Hospital, The Air Force Military Medical University, Xi’an 710032, China; 3 Department of Pathology, The First Affiliated Hospital of the Army Medical University, Chongqing 400038, China

**Keywords:** prostate cancer, neuroendocrine tumors, volume-regulated anion channel, leucine-rich repeat protein, cisplatin

## Abstract

Neuroendocrine prostate cancer (NEPC) is a lethal subtype of prostate cancer (PCa) that emerges under androgen deprivation and is associated with therapeutic resistance. The contribution of volume-regulated anion channels (VRACs) to this process remains poorly understood. This study identified leucine-rich repeat-containing 8 subunit D (LRRC8D), a VRAC subunit, as the only family member consistently downregulated in NEPC and associated with neuroendocrine (NE)-like features. LRRC8D downregulation was accompanied by suppression of swelling-activated VRAC currents, increased synaptophysin (SYP) expression, decreased cisplatin sensitivity, and neurosecretory remodeling. Conversely, LRRC8D overexpression enhanced cisplatin-induced apoptosis, reduced colony formation, and suppressed tumor growth in xenograft models, including under cisplatin treatment. Consistent alterations in LRRC8D and SYP expression were also observed in enzalutamide-resistant patient-derived organoids. Mechanistically, RE1-silencing transcription factor (REST) promoted LRRC8D transcription. Functional analyses further demonstrated that CAV-1 acted upstream of LRRC8D, and LRRC8D negatively regulated STAT3 activation. Together, these findings indicate that LRRC8D influences PCa phenotype and platinum responsiveness, and implicate a regulatory axis involving LRRC8D and CAV-1/STAT3 signaling in NE-associated features of advanced PCa. Functional analyses further showed that CAV-1 acted upstream of LRRC8D, and LRRC8D negatively regulated STAT3 activation. Together, these findings indicate that LRRC8D influences PCa phenotype and platinum responsiveness and implicate a regulatory axis involving LRRC8D and CAV-1/STAT3 signaling in NE-associated features of advanced PCa.

## 1. Introduction

Neuroendocrine prostate cancer (NEPC) represents one of the most lethal phenotypes of prostate cancer (PCa). Unlike the conventional adenocarcinoma subtype that relies on androgen receptor (AR) signaling, NEPC commonly emerges under therapeutic pressure and displays resistance to nearly all AR-directed strategies [1]. Clinically, NEPC accounts for approximately 10% of castration-resistant PCa (CRPC) and is associated with a median survival of less than one year [2,3]. Patients often present with visceral metastases, rapid disease progression, and poor responses to hormonal therapies [4]. Although platinum-based chemotherapy can provide transient benefit in a subset of cases, effective therapeutic strategies for NEPC remain scarce [5], underscoring the urgency to identify molecular determinants of drug response and resistance in this aggressive disease.

NEPC is characterized by the loss of AR signaling and the induction of canonical neuroendocrine (NE) markers, including synaptophysin (SYP), chromogranin A (CHGA), and neuron-specific enolase (NSE). Serine/arginine repetitive matrix 4 (SRRM4) overexpression, cyclic adenosine monophosphate (cAMP) elevation, and prolonged exposure to AR inhibitors such as enzalutamide (ENZ) can induce NE-like phenotypes in adenocarcinoma models [6,7,8]. However, in vivo NEPC development is a multifactorial process involving transcriptional reprogramming, tumor suppressor loss, and epigenetic remodeling, and the precise regulatory events remain incompletely defined [9]. Several factors, such as POU class 3 homeobox 2 (BRN2) and RE1-silencing transcription factor (REST), have been implicated as transcriptional regulators of NE lineage plasticity [10,11]. Nevertheless, mechanisms beyond transcriptional control may also contribute to this phenotypic transition.

Ion channels, which regulate cellular signaling, metabolite transport, and drug uptake, are increasingly recognized as druggable targets [12], representing a potential but largely unexplored regulatory layer in NEPC. Among these, volume-regulated anion channels (VRACs) constitute a major class of anion channels involved in cellular osmotic balance and metabolite transport. VRACs are formed by heteromeric assemblies of leucine-rich repeat-containing 8 (LRRC8) family members (A–E) and regulate diverse processes, including apoptosis, migration, and tumor growth [13,14]. Among VRAC subunits, leucine-rich repeat-containing 8 subunit D (LRRC8D) is of particular interest because it uniquely governs the uptake of platinum-based agents such as cisplatin and carboplatin [13]. Clinically, NEPC displays resistance to AR-targeted therapies but retains relative sensitivity to platinum agents [15], suggesting that alterations in LRRC8D-mediated transport could link chemotherapy response to lineage plasticity. Despite these functions, whether LRRC8D directly couples platinum sensitivity with NE differentiation remains unknown.

This study aimed to address this gap by focusing on LRRC8D as a candidate regulator of PCa progression and therapeutic response. Using cAMP-induced and SRRM4-overexpressing cellular models, NE-like cell lines, patient-derived organoids, and xenograft models, the study integrated bioinformatic, electrophysiological, structural, and functional analyses to examine the role of LRRC8D in PCa phenotype and platinum sensitivity. The findings identified LRRC8D as a previously unrecognized regulator with NE-like features and platinum responsiveness in PCa, highlighting its potential relevance to therapy resistance and treatment strategies in advanced disease.

## 2. Materials and Methods

### 2.1. Transcriptomic Data Sources

To evaluate LRRC8D expression across different PCa states, multiple transcriptomic datasets covering adenocarcinoma, CRPC, and NEPC were analyzed. GSE239593 (transcriptomic profiles of engineered LNCaP vs. PC-3 tissue constructs from the GEO database) [16], GSE266283 (RNA-seq data from LNCaP and androgen-independent LNCaP-AI cells) [17], GSE59986 (LTL331 PDX series at different NEPC stages) were analyzed [18].

For clinical validation, GSE21034 (primary and metastatic PCa samples with clinical annotation) [19] and single-cell RNA-seq (scRNA-seq) data from GSE137829 were used to evaluate the relationship between LRRC8D and SYP at the cellular level [20]. Independent validation was performed using metastatic CRPC/NEPC cohorts available in cBioPortal (https://www.cbioportal.org/), (accessed on 1 April 2019) including the Beltran 2016 dataset (114 metastatic samples from 81 patients) [21] and the Broad/Cornell 2012 dataset [22].

For analysis of VRAC subunits, GSE105033 was examined. This dataset contains microarray profiles from NCI-H660 NEPC cells with HP1α knockdown or control and V16D adenocarcinoma cells under HP1α overexpression or NE-inducing conditions (13 samples in total) [23]. Additional PCa transcriptomic data were obtained from the Oncomine database (https://www.oncomine.org/) (accessed on 15 December 2018) [11,19].

All expression values were normalized according to the original study protocols, and correlation with NE markers was assessed using Pearson or Spearman’s correlation based on the data distribution.

### 2.2. Cell Lines

PCa cell lines (LNCaP, 22RV1, C42, PC3, DU145, and NCI-H660) were obtained from the American Type Culture Collection (ATCC, Manassas, VA, USA). All cell lines were authenticated and confirmed to be free of mycoplasma contamination. Except for NCI-H660, all lines were cultured in RPMI 1640 supplemented with 10% fetal bovine serum (FBS; Gibco, Grand Island, NY, USA). NCI-H660 cells were cultured in RPMI 1640 with 5% FBS, supplemented with sodium selenite, beta-estradiol, insulin, hydrocortisone, transferrin, and L-glutamine (Sigma-Aldrich, St. Louis, MO, USA).

### 2.3. Construction and Identification of NE-like Cell Models

NE-like PCa cell models were constructed by lentiviral overexpression of SRRM4 in LNCaP and 22RV1 cells. The coding sequence of SRRM4 (GenBank accession number: NM_194286) was synthesized and verified by Sanger sequencing. Lentiviral packaging and infection were performed as previously described [24]. Stable overexpression of SRRM4 was established by puromycin selection (2 μg/mL for 7 days). Overexpression was confirmed by quantitative real-time PCR (qRT-PCR) and Western blotting.

### 2.4. Animal Experiments

Male BALB/c athymic nude mice (RRID:MGI:2683685) (6–7 weeks old) were purchased from Beijing Vitalstar Biotechnology (Beijing, China) and housed under specific-pathogen-free conditions at the Laboratory Animal Center of the Air Force Military Medical University (AMMU; Xi’an, China). ARRIVE guidelines were followed for all animal experiments. All procedures were approved by the Institutional Animal Care and Use Committee of AMMU (protocol No. 20190207) and conducted in accordance with the Guide for the Care and Use of Laboratory Animals (NIH Publications No. 8023, revised 1978).

Mice were anesthetized with isoflurane during surgery and imaging. One week after bilateral castration, mice were randomized into two groups (22RV1-NC or 22RV1-SRRM4) and subcutaneously injected with 1 × 10^6^ cells [25]. 22RV1-NC cells served as a CRPC control, whereas 22RV1-SRRM4 cells represented an NE-like model. Tumor growth was monitored to compare growth kinetics between CRPC and NE-like tumors. Tumor dimensions and body weight were measured every 3 days. Tumor volume (V) was calculated as V = 1/2 × (W^2^ × L). Mice were sacrificed when tumor volume reached 1000 mm^3^. Xenograft fragments (~100 mm^3^) were serially transplanted into newly castrated mice for six generations, with the initial engraftment defined as passage 0 (P0) and the final as passage 6 (P6). Remaining tissues were snap-frozen or processed for immunohistochemistry (IHC).

To assess the functional role of LRRC8D in NE-like tumors, 22S8DNC or 22S8DOE cells (SRRM4-overexpressing cells with or without LRRC8D overexpression) were subcutaneously injected into castrated mice. Tumor growth was monitored to determine the impact of LRRC8D on NE-like tumor progression.

To evaluate cisplatin responsiveness, 22S8DNC or 22S8DOE cells were implanted into mice using the same procedure. When tumor volume reached approximately 300 mm^3^, cisplatin (1 mg/kg; CS1535, G-Clone, Beijing, China) was administered via intraperitoneal injection. Tumor growth was monitored to assess treatment response in the different cell-derived xenograft (CDX) models. Sample sizes for in vivo experiments (typically *n* = 3–6 per group) were determined according to established practice for comparable CDX studies in this field [26].

### 2.5. Cell Viability Analysis

Cells were seeded at a density of 1 × 10^4^ per well in 96-well plates and treated using ENZ (10 μM; Selleck Chemicals, Houston, TX, USA) or cisplatin (3 μM; Selleck Chemicals) for up to 8 days. Cell viability was determined at the indicated time points using the cell counting kit-8 (7Sea Biotech, Shanghai, China) according to the manufacturer’s instructions. Optical density (OD) was measured at 450 nm using a microplate reader (Bio-Tek Instruments, Winooski, VT, USA).

### 2.6. qRT-PCR

Cells (2 × 10^5^ per well) were seeded in 6-well plates and harvested using 1 mL TRIzol reagent (Invitrogen, Thermo Fisher Scientific, Waltham, MA, USA). Total RNA was extracted according to the manufacturer’s protocol, and cDNA was synthesized using the PrimeScript™ RT Reagent Kit (Takara, Beijing, China). qRT-PCR was performed using TB Green^®^ Fast qPCR Mix (Takara) on a StepOnePlus Real-Time PCR Detection System (Applied Biosystems, Thermo Fisher Scientific). Relative mRNA expression was calculated using the 2^−ΔΔCt^ method, with β-actin used as the internal control. Primer sequences are provided in Supplementary Table S1.

### 2.7. Western Blot Analysis

Western blot analyses were performed using standard protocols [27]. Primary antibodies included anti-REST (22242-1-AP; Proteintech, Wuhan, China), anti-LRRC8D (104245-T32; Sino Biological, Beijing, China), anti-CHGA (ab45179; Abcam, Cambridge, UK), anti-NSE (30394; ProMab Biotechnologies, Changsha, China), anti-β-actin (AT0001; Engibody, Shanghai, China), anti-SYP (ab32127; Abcam), anti-caspase-3 (9662S; CST, Danvers, MA, USA), anti-cleaved caspase-3 (9661; CST), Bax (41300S; CST, Danvers, MA, USA), anti-caveolin-1 (CAV-1; 3267; CST), anti-STAT3 (T55292; Thermo Fisher Scientific), and anti-phospho-STAT3 (T56566; Thermo Fisher Scientific). Chemiluminescent signals were detected using a G:BOX Syngene imaging system (Syngege, Cambridge, UK). Band intensities were quantified using Image J 1.48v (NIH, Bethesda, MD, USA).

### 2.8. IHC and Cellular Immunocytochemistry

A total of 16 PCa tissue specimens of patients diagnosed with HNPC, CRPC, or NEPC were obtained from the Department of Pathology, Xijing Hospital (Xi’an, China) and subjected to IHC analysis. Samples were fixed in 4% paraformaldehyde, embedded in paraffin, and sectioned at 5 μm thickness. All procedures involving human tissue were approved by the Clinical Research Ethics Board of Xijing Hospital (approval No. KY20193035). For cellular immunocytochemistry, coverslips were pre-plated in 6-well plates, and 2 × 10^5^ cells were seeded overnight before fixation with 4% paraformaldehyde.

Primary antibodies used in IHC include anti-SYP (ab32127; Abcam), anti-prostate-specific antigen (PSA; ab76113; Abcam), anti-LRRC8A (orb185053; Biobyt, Cambridge, UK), and anti-LRRC8D (orb185056; Biobyt). A biotin-labeled goat anti-rabbit secondary antibody (Kangwei, Beijing, China) was used as previously described [28]. DAB staining and counterstaining were performed using standard methods. IHC and cellular immunocytochemistry images were captured using a microscope (Olympus, Tokyo, Japan). Staining intensity was quantified in three randomly selected fields per section/sample using ImageJ software 1.48v.

### 2.9. Patch-Clamp Recording

Whole-cell voltage-clamp recordings were conducted to measure swelling-activated chloride currents (I_Cl,swell_) using an Axon patch 200B amplifier (Axon Instruments, Foster City, CA, USA) as previously described [29]. Isotonic and hypotonic bath solutions, as well as internal pipette solutions, were prepared according to established protocols [29]. Tetrodotoxin (8 μM) and nifedipine (5 μM) were included in the bath to block voltage-gated Na^+^ and L-type Ca^2+^ channels, respectively. cAMP was added to both isotonic and hypotonic bath solutions to induce I_Cl,swell_. Current amplitudes were measured 5 ms after the onset of voltage step pulses, and current density at +100 mV was used for quantification.

### 2.10. Colony Formation Assay

A standard clonogenic assay was performed to assess the long-term proliferative potential of tumor cells following various treatments. In brief, cells were seeded at 500 cells/well in 6-well plates and cultured for 7–14 days to allow for colony formation. Colonies were fixed with paraformaldehyde, stained with crystal violet, manually counted, and photographed using a digital camera (Canon, Tokyo, Japan) under standardized lighting conditions. The experiment was performed in triplicate, and the colony formation rate was calculated and compared across groups.

### 2.11. SiRNA Transfection

CAV-1 siRNA was provided by Hanbio Biotechnology Co., Ltd. (Shanghai, China). LNCaP cells at approximately 75% confluence were transfected with 50 µM siRNA using Lipofectamine (Thermo Fisher Scientific). After 48 h of incubation, the cells were harvested for qRT-PCR and Western blot analysis.

### 2.12. Flow Cytometry

Following treatment with ENZ (10 μM) or cisplatin (10 μM) for 12 h, apoptosis was analyzed by flow cytometry. Cells were seeded at 1 × 10^6^ per flask, with one unstained control for compensation and the remaining flasks subjected to drug treatment. Both floating and adherent cells were collected, washed with PBS, and stained using an Annexin V-PE/7-AAD apoptosis detection kit (Solarbio, Beijing, China). Because the cells expressed GFP, PE/7-AAD staining was used to avoid spectral overlap. Total apoptosis was defined as the sum of early and late apoptotic populations.

### 2.13. Construction and Culture of Patient-Derived Organoids (PDOs)

Fresh PCa specimens (~1 cm^3^) were obtained immediately after surgery and divided into three portions: the majority for organoid culture, a small portion fixed in 4% paraformaldehyde, and the remainder stored at −80 °C. Tissues were minced and digested in DMEM/F12 containing 1.5 mg/mL collagenase type II (Invitrogen), 10 μg/mL hyaluronidase type IV (Invitrogen), and 10 μM Y-27632 (Sigma-Aldrich) at 37 °C with agitation until complete dissociation. Digestion was terminated with 10% serum-containing medium. The suspension was filtered through a 100 μm strainer, centrifuged (800–1200 rpm, 3 min, 4 °C), and washed with PBS. Cell pellets were resuspended in 70% Matrigel (Hyclone) and 30% DMEM/F12, and 30 μL droplets were seeded into 24-well plates. After solidification at 37 °C for 15–30 min, 500 μL organoid culture medium was added. Medium was changed every 3 days, and organoids were passaged every 1–2 weeks by mechanical dissociation or TrypLE digestion at a 1:2–1:4 ratio. For cryopreservation, organoids (~100 μm diameter) were collected and resuspended in 90% serum and 10% DMSO, stored at −80 °C for 24 h, and then transferred to liquid nitrogen. For recovery, samples were rapidly thawed at 37 °C and processed as in primary culture.

### 2.14. Induction of PDO Drug Resistance and Drug Sensitivity Assay

ENZ resistance was established in PDOs using a stepwise dose-escalation protocol. Treatment was initiated at 5 μM ENZ, and the concentration was gradually increased according to organoid growth status until a maintenance concentration of 25 μM was reached. Organoids were cultured under continuous exposure until stable growth was observed at 25 μM ENZ, generating ENZ-resistant PDOs (PDO^ENZR^). For drug sensitivity testing, PDOs and PDO^ENZR^ were dissociated into single cells, counted, and resuspended in organoid medium containing 10% Matrigel. Cells were seeded into 96-well plates at 3500 cells per well. After embedding, organoids were treated with graded concentrations of ENZ (6.25, 12.5, 25, 50, and 100 μM) and incubated at 37 °C for 72 h prior to viability assessment.

### 2.15. Transmission Electron Microscopy (TEM)

Adherent cells were harvested by enzymatic digestion and centrifuged for 10 min. Cells were fixed in a solution containing 4% paraformaldehyde and 0.3% glutaraldehyde, followed by thorough washing, graded acetone dehydration, and embedding in LR White resin (Sigma-Aldrich). Ultrathin sections (50–70 nm) were mounted on nickel grids and sequentially stained with uranyl acetate and lead citrate. Imaging was performed using an EM 109 R electron microscope (Zeiss, Oberkochen, Germany).

### 2.16. Dual-Luciferase Reporter Gene Assay

Potential REST-binding sites within the LRRC8D promoter were predicted using JASPAR (http://jaspar.binf.ku.dk/) (accessed on 15 October 2020). A wild-type (Wt) sequence (5′-TCTCTGTTCATGTTCCTGT-3′) upstream of the LRRC8D promoter (NM_001134479) and a mutant (Mut) version (5′-ACACAGATGAAGATGCAGA-3′; A → T, C → G substitutions) were synthesized and cloned into the pGL3-basic vector (Hanbio, Shanghai, China). The full-length REST coding sequence was subcloned into the pcDNA3.1 vector. HEK293T cells were seeded in 24-well plates and co-transfected at ~70–80% confluence with 400 ng of Wt-LRRC8D or Mut-LRRC8D reporter, 200 ng of REST overexpression plasmid, and 200 ng of Renilla PRL plasmid using Lipofectamine 2000 (Thermo Fisher Scientific). Luciferase activity was measured using the Dual-Luciferase^®^ Reporter Assay System (Promega, Beijing, China) and normalized to Renilla luciferase signal.

### 2.17. Immunofluorescence (IF)

LNCaP cells grown on coverslips were fixed with 4% paraformaldehyde for 10 min at room temperature, permeabilized with 0.05% Triton X-100 for 10 min and blocked with BSA (5%) for 1 h at room temperature. Subsequently, cells were incubated with primary antibodies against LRRC8D (orb185056; Biobyt), REST ((22242-1-AP; Proteintech) overnight at 4 °C in the dark. Subsequently, samples were incubated with appropriate fluorophore-conjugated secondary antibodies (GB25303, GB28303, Servicebio, Wuhan, China) for 1 h at room temperature in the dark. Cell nuclei were counterstained with DAPI. Images were captured using a microscope (Olympus, Japan).

### 2.18. Co-Immunoprecipitation (co-IP)

22RV1 cells (1 × 10^7^) were lysed, and the supernatants were incubated with anti–CAV-1 antibody–coupled agarose beads (#3267; Cell Signaling Technology, Danvers, MA, USA) in IP buffer. After washing, the immunoprecipitated protein complexes were subjected to SDS-PAGE and analyzed by Western blotting. IgG was used as a negative control. Protein signals were visualized and quantified using a G:BOX imaging system (Syngege).

### 2.19. Molecular Docking

Molecular docking was carried out as previously described [30]. The X-ray crystal structures of LRRC8D (6M04) and CAV1 (6SC0) were retrieved from the Protein Data Bank (http://www.rcsb.org/pdb/home/home.do) (accessed on 10 December 2022). Docking Web Server (GRAMM) was used for protein-protein docking. The resulting protein-protein complex was manually optimized by removing water and adding polar hydrogen. Finally, the protein–protein interactions were predicted, and the protein–protein interaction figure was generated by PyMOL 3.0. The LRRC8D protein was visualized as a slate cartoon model, and CAV-1 was displayed as a cyan cartoon model. Binding residues were shown as stick representations in the corresponding colors. For detailed visualization, the binding interface was presented in the context of the respective protein structure.

### 2.20. MQAE Assay

N-(Ethoxycarbonylmethyl)-6-methoxyquinolinium bromide (MQAE, HY-D0090, MedChemExpress, Monmouth Junction, NJ, USA) was used to assess Cl^−^ concentrations in cells. The fluorescence of MQAE is quenched collisionally upon interaction with Cl^−^, resulting in a Cl^−^ concentration-dependent decrease in fluorescence intensity [31]. 22S-8DNC (control), 22S8DKD (22S cells with LRRC8D knockdown), 22S8DOE (LRRC8D-overexpressing) cells were incubated with the halide-sensitive fluorescent dye MQAE for 1 h. After hypotonic bath solutions were added to cells, the intracellular Cl^−^ ions decreased and MQAE fluorescence increased. Fluorescence was then immediately captured using a laser confocal microscope (Zeiss LSM 710, Carl Zeiss, Jena, Germany) (excitation: 350 nm; emission: 460 nm).

### 2.21. Gene Ontology (GO) Enrichment Analysis

For GO enrichment analysis, differentially expressed genes (DEGs) between LRRC8D-overexpressing cells and control cells were identified based on transcriptomic profiling. GO biological process annotation and enrichment were performed using the clusterProfiler R package. Significantly enriched terms were determined using a hypergeometric test with a false discovery rate (FDR) cutoff of <0.05. Results were visualized using Sankey and bubble plots.

### 2.22. Statistical Analysis

All data were analyzed using GraphPad Prism 10.3.1 (Dotmatics, Boston, MA, USA). Data were expressed as the mean ± standard deviation (SD). Statistical significance was assessed using Student’s *t*-test, multiple t-test, or one-way ANOVA. A *p*-value < 0.05 was considered statistically significant. All quantitative analyses were performed by an investigator blinded to the group allocation.

## 3. Results

### 3.1. NE-Associated States Exhibit Reduced LRRC8D Expression and Diminished VRAC Activity

**Figure 1:**
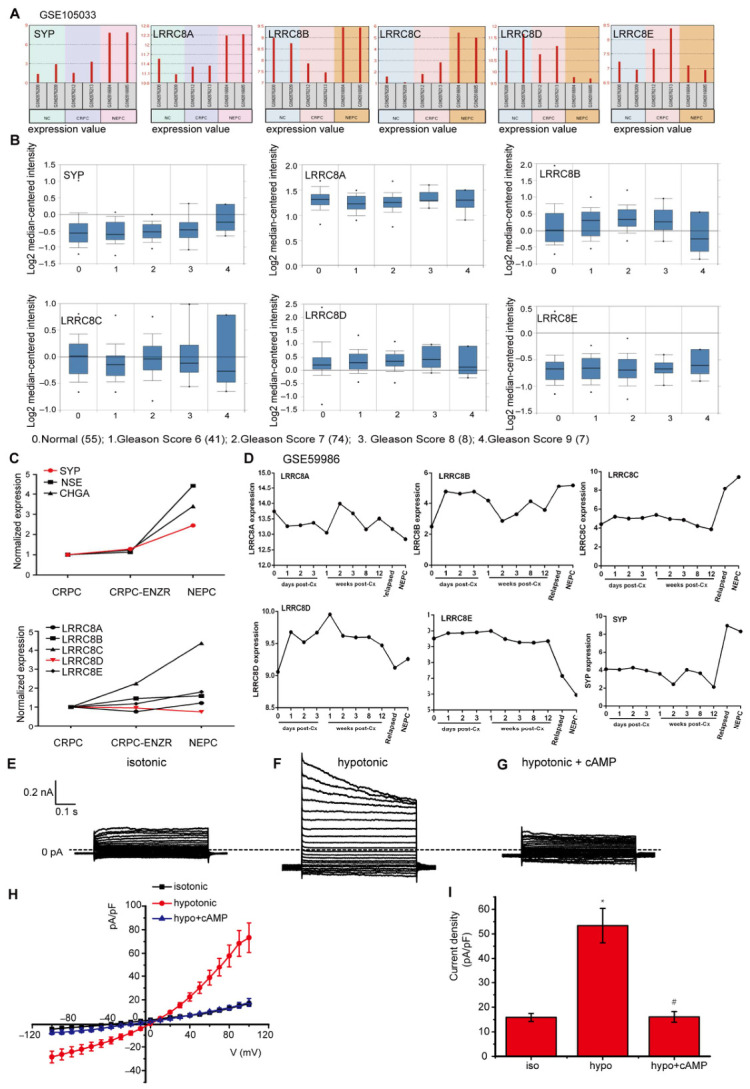
Patch-clamp recording of swelling-activated chloride currents (I_Cl,swell_) in LNCaP cells under isotonic, hypotonic, and hypotonic plus cAMP conditions. (**A**). Expression profiles of LRRC8A–E and SYP in the GSE105033 microarray dataset. (**B**). Expression levels of LRRC8A–E and SYP in the GSE21034 prostate cancer (PCa) dataset. Dots represent maximum and minimum outliers from the main data set. (**C**). Expression levels of LRRC8A–E and SYP in an independent prostate cancer dataset reported by Bishop et al. (**D**). Time-course expression of LRRC8D and SYP in the GSE59986 dataset, including post-castration (post-Cx), relapse, and terminal NEPC stages. (**E**). Representative whole-cell current traces recorded under isosmotic conditions. (**F**). Current traces recorded under hypotonic stimulation. (**G**). Current traces recorded under hypotonic conditions with 2 μM cAMP treatment. (**H**). Current–voltage (I–V) relationships derived from the recordings shown in panels (**E**–**G**). (**I**). Quantification of current densities at +100 mV. Experimental groups include isotonic (iso), hypotonic (hypo), and hypotonic plus cAMP (hypo + cAMP). * *p* < 0.05 vs. isotonic, ^#^ *p* < 0.05 vs. hypotonic; n = 5.

To determine whether VRAC subunits are associated with NE phenotypes in PCa, expression profiles of LRRC8A–LRRC8E were analyzed together with the NE marker SYP across multiple independent transcriptomic datasets. In the GSE105033 cohort, LRRC8D expression decreased as SYP expression increased across disease progression, whereas other LRRC8 subunits did not display a consistent inverse trend (Figure 1A). A similar relationship was observed in the GSE21034 dataset (Figure 1B). Analysis of an independent PCa cohort reported by Bishop et al. [11] confirmed this inverse correlation (Figure 1C). Longitudinal evaluation of GSE59986 revealed progressive reduction in LRRC8D expression during post-castration progression, accompanied by increased SYP expression in relapse and terminal NEPC stages (Figure 1D). These findings indicate that LRRC8D downregulation is reproducibly associated with NE marker enrichment in clinical contexts.

Given the consistent reduction in LRRC8D in NE-associated clinical datasets, VRAC functional activity was examined under conditions that induce NE-like features in vitro. Whole-cell patch-clamp recordings in LNCaP cells revealed minimal basal chloride currents under isotonic conditions (Figure 1E). Hypotonic stimulation elicited a characteristic I_Cl,swell_ (Figure 1F), confirming VRAC activation. cAMP treatment for NE induction significantly suppressed the hypotonicity-induced current (Figure 1G). The corresponding current-voltage relationship demonstrated reduced outward conductance in the hypotonic plus cAMP condition (Figure 1H), and quantification at +100 mV confirmed significant attenuation of I_Cl,swell_ (Figure 1I). These results indicate that NE-inducing conditions are associated with functional suppression of VRAC-mediated chloride conductance, consistent with the clinical observation of LRRC8D downregulation.

Consistently, SRRM4-overexpressing NE-like cells exhibited reduced I_Cl,swell_ compared with control cells (Figure S1A). Analysis of PCa datasets curated in Oncomine confirmed decreased LRRC8D mRNA expression in NE-associated settings (Figure S1B). NE-like induction by cAMP treatment or SRRM4 overexpression was confirmed by increased SRRM4, SYP, and CHGA expression at both mRNA and protein levels (Figure S1C–E). In contrast, LRRC8A knockdown in 22RV1 cells did not alter CHGA, SYP, or NSE protein expression (Figure S1F–G), supporting a subunit-specific association of LRRC8D with NE-related marker regulation. Together, these data indicate that LRRC8D reduction and VRAC suppression are coordinated features of NE-associated states.

### 3.2. Reduced LRRC8D Expression Characterizes NE-Associated States and Inversely Correlates with NE Marker Expression

**Figure 2:**
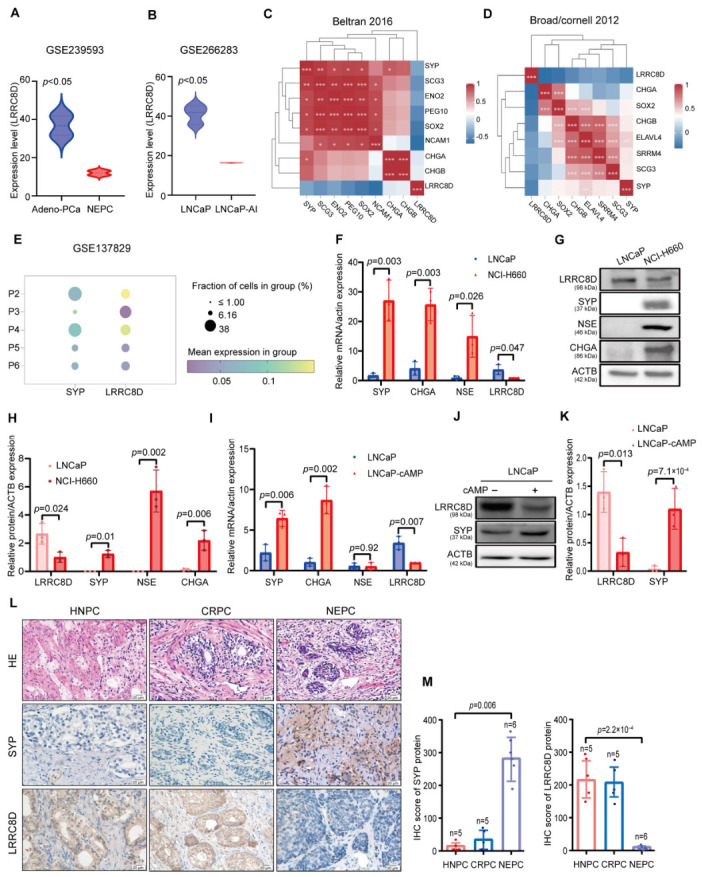
LRRC8D and SYP expression profiles in PCa and neuroendocrine (NE)-associated models. (**A**). LRRC8D mRNA expression in adenocarcinoma prostate cancer (Adeno-PCa) and neuroendocrine prostate cancer (NEPC) samples from the GSE239593 dataset (n = 2/group). (**B**). LRRC8D mRNA expression in LNCaP and LNCaP-AI cells from the GSE266283 dataset (n = 3/group). (**C**). Correlation matrix of LRRC8D and NE signature genes in the Beltran 2016 [21] cohort. (**D**). Correlation matrix of LRRC8D and NE signature genes in the Broad/Cornell 2012 cohort. (**E**). Single-cell expression of LRRC8D and SYP in NEPC patient samples (P2, P3, P4, P5, P6) from the GSE137829 dataset. (**F**). Relative mRNA expression of Syp, Chga, Nse, and Lrrc8D in NCI-H660 NEPC cells compared with LNCaP adenocarcinoma cells (n = 3). (**G**). Representative immunoblot of LRRC8D, SYP, NSE, CHGA, and β-ACTIN in NCI-H660 and LNCaP cells. (**H**). Quantification of LRRC8D, SYP, NSE, and CHGA protein expression in NCI-H660 and LNCaP cells (n = 3). (**I**). Relative mRNA expression of Syp, Chga, Nse, and Lrrc8d in LNCaP cells with or without cAMP treatment (n = 3). (**J**). Representative immunoblot of LRRC8D, SYP, and β-ACTIN in LNCaP cells with or without cAMP treatment. (**K**). Quantification of SYP and LRRC8D protein expression in LNCaP cells with or without cAMP treatment (n = 3). (**L**). H&E and immunohistochemical (IHC) staining of LRRC8D and SYP in tissues from patients with hormone-naïve prostate cancer (HNPC), castration-resistant prostate cancer (CRPC), and NEPC. Scale bar: 20 μm. (**M**). IHC scores of SYP and LRRC8D in the indicated groups. All experiments were repeated three times. Data are presented as the mean ± SD. *p* < 0.05 was considered statistically significant. “*” represented *p* < 0.05, “**” represented *p* < 0.01, “***” represented *p* < 0.001.

To further assess the relationship between LRRC8D expression and NE-associated features, LRRC8D levels were systematically examined across independent clinical cohorts and experimental models. Public transcriptomic analyses demonstrated that LRRC8D mRNA was significantly reduced in clinical NEPC samples compared with adenocarcinoma samples (GSE239593; Figure 2A), as well as in NE-like LNCaP-AI cells versus parental LNCaP cells (GSE266283; Figure 2B). Correlation analyses of the Beltran 2016 [21] and Broad/Cornell 2012 cohorts further revealed a consistent inverse relationship between LRRC8D and canonical NE markers, particularly SYP (Figure 2C,D). scRNA-seq analysis (GSE137829) supported this pattern, which showed lower LRRC8D expression in advanced, SYP-enriched NEPC clusters (Figure 2E). These findings indicate that the inverse association between LRRC8D and NE markers is maintained across bulk and single-cell transcriptomic datasets.

This inverse relationship was also validated in experimental models and clinical specimens. The NEPC cell line NCI-H660 exhibited lower LRRC8D expression and higher NE marker levels compared with LNCaP adenocarcinoma cells (Figure 2F–H). Similarly, cAMP-treated LNCaP cells showed reduced LRRC8D expression accompanied by increased SYP expression at both mRNA and protein levels (Figure 2I–K). In clinical PCa samples, IHC analysis demonstrated reduced LRRC8D staining and elevated SYP expression in NEPC compared with HNPC and CRPC tissues (Figure 2L,M). Collectively, these data indicate that LRRC8D downregulation is a recurrent feature of NE-associated PCa and is consistently inversely correlated with canonical NE marker expression across transcriptomic, cellular, and tissue levels.

### 3.3. LRRC8D Reduction Accompanies NE Marker Enrichment and Accelerated Tumor Growth in SRRM4-Driven Models

**Figure 3:**
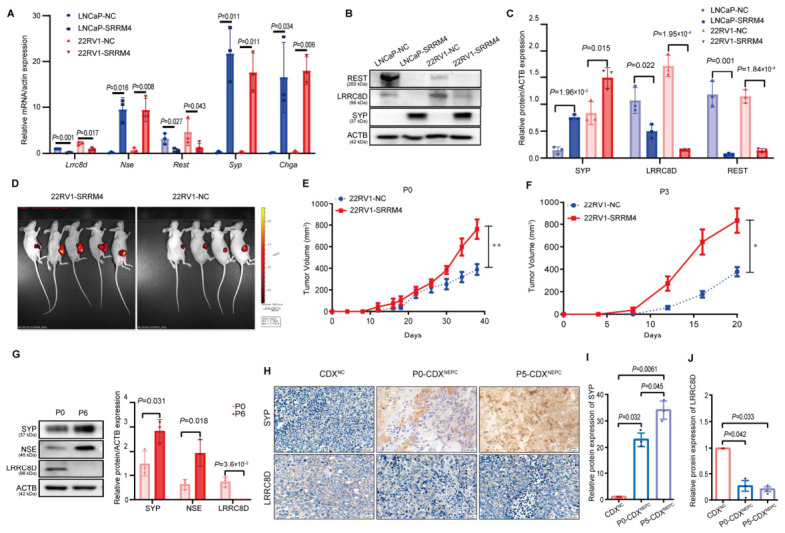
LRRC8D is downregulated in SRRM4-induced NE-like cells and cell-derived xenograft (CDX) models. (**A**). Relative mRNA expression of Lrrc8d, Nse, Rest, Syp, and Chga in LNCaP and 22RV1 cells transduced with SRRM4. (**B**). Representative immunoblot of REST, LRRC8D, SYP, and β-ACTIN in SRRM4-overexpressing LNCaP and 22RV1 cells. (**C**). Quantification of REST, LRRC8D, and SYP protein expression in the indicated groups (n = 3). (**D**). In vivo imaging showing accelerated growth of 22RV1-SRRM4 xenografts (n = 5) compared with 22RV1-NC controls (n = 4). (**E**). Tumor growth curves of 22RV1-SRRM4 (n = 5) and 22RV1-NC (n = 4) xenografts. (**F**). Third-generation xenografts exhibited earlier onset and faster growth than first-generation xenografts (n = 5). (**G**). Representative immunoblot of SYP, NSE, LRRC8D, and β-ACTIN in tumor tissues from first-(P0) and sixth-generation (P6) xenografts derived from 22RV1-SRRM4 cells (n = 3). (**H**). Immunohistochemical staining of SYP and LRRC8D in tumor tissues from control 22RV1 xenografts (CDX^NC^), first-generation 22RV1-SRRM4 xenografts (P0-CDX^NEPC^), and fifth-generation 22RV1-SRRM4 xenografts (P5-CDX^NEPC^). Scale bar: 20 μm. (**I**). Quantification of SYP protein expression in the indicated groups (n = 3). (**J**). Quantification of LRRC8D protein expression in the indicated groups (n = 3). “*” represented *p* < 0.05, “**” represented *p* < 0.01.

To assess the functional relevance of LRRC8D reduction in NE-like contexts, SRRM4-overexpressing PCa models were examined. SRRM4 overexpression in LNCaP and 22RV1 cells resulted in increased expression of NE markers, including SYP, CHGA, and NSE, accompanied by reduced REST and decreased LRRC8D expression at both mRNA and protein levels (Figure 3A–C). In vivo, 22RV1-SRRM4 xenografts exhibited significantly accelerated tumor growth compared with control xenografts at both P0 and P3 passages (Figure 3D–F). Serial passaging of SRRM4-derived xenografts demonstrated progressive enrichment of NE markers, together with further reduction in LRRC8D expression (Figure 3G). IHC analyses confirmed increased SYP staining and diminished LRRC8D expression in CDX^NEPC^ tumors relative to controls (Figure 3H–J). These data indicate that LRRC8D downregulation accompanies NE marker enrichment and enhanced tumor growth in SRRM4-driven models.

### 3.4. LRRC8D Restrains Neurosecretory Remodeling and Tumor Growth in NE-like Models

**Figure 4:**
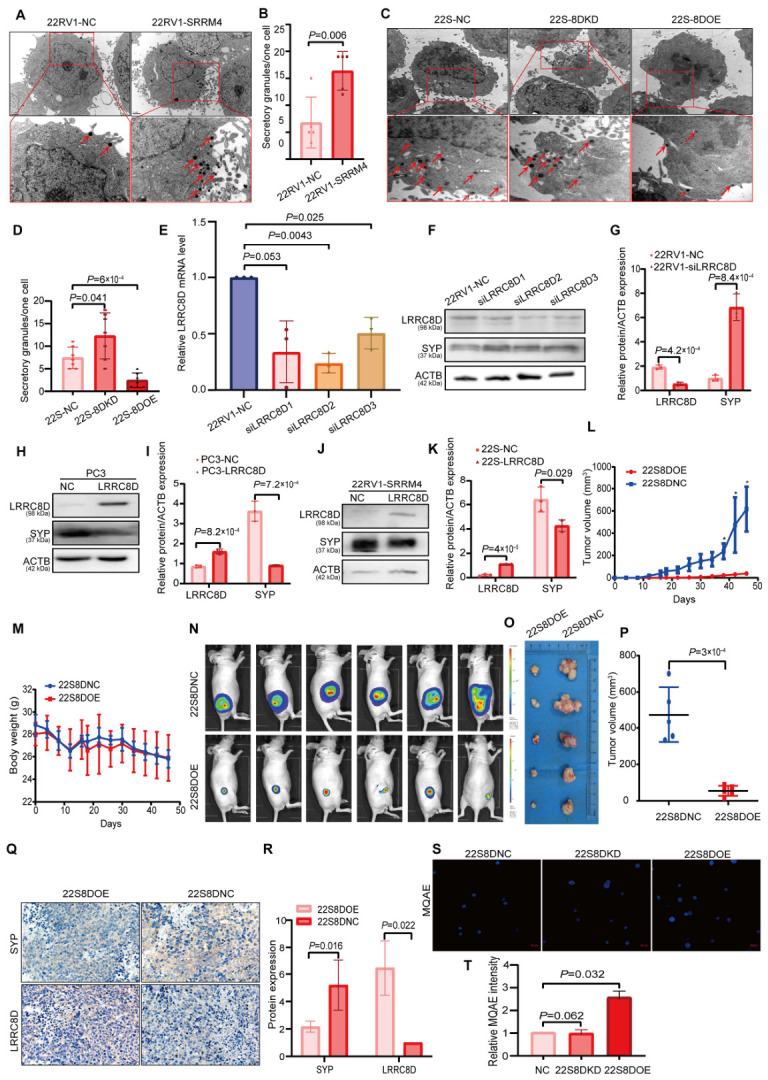
LRRC8D restrains neurosecretory granule formation and tumor growth in NE-like models. (**A**). Transmission electron microscopy of 22RV1-SRRM4 cells (22S) and control 22RV1-NC cells. Red boxes indicate magnified regions; red arrows denote neuroendocrine granules near the plasma membrane. (**B**). Quantification of secretory granules per cell in the indicated groups (n = 5). (**C**). Transmission electron microscopy of 22S-NC, LRRC8D-knockdown (22S-8DKD), and LRRC8D-overexpressing (22S-8DOE) cells. (**D**). Quantification of secretory granules per cell in the indicated groups (n = 9). (**E**). Relative LRRC8D mRNA expression following LRRC8D knockdown. (**F**). Representative immunoblot of LRRC8D and SYP in 22RV1-NC cells after LRRC8D knockdown. (**G**). Quantification of LRRC8D and SYP protein expression in the indicated groups (n = 3). (**H**). Representative immunoblot of LRRC8D and SYP in PC3 cells overexpressing LRRC8D and corresponding controls. (**I**). Quantification of LRRC8D and SYP protein expression in the indicated groups (n = 3). (**J**). Representative immunoblot of LRRC8D and SYP in 22S cells overexpressing LRRC8D and corresponding controls. (**K**). Quantification of LRRC8D and SYP protein expression in the indicated groups (n = 3). (**L**). Tumor growth curves of nude mice bearing xenografts (n = 6). (**M**). Body weight monitoring of nude mice bearing xenografts (n = 6). (**N**). In vivo bioluminescence imaging of xenograft-bearing mice. (**O**). Representative images of excised xenograft tumors (n = 5). (**P**). Quantification of tumor volumes after excision (n = 5). (**Q**,**R**). Immunohistochemical staining and quantification of LRRC8D and SYP expression in xenograft tissues shown in panel O. Scale bar: 20 μm. (**S**). Measurement of Cl^−^ expression in 22S8DNC, 22S8DKD, and 22S8DOE cells using MQAE (10 μM, 1 h) (scale bar = 20 μm). (**T**). Relative MQAE intensity in Figure 4S. Note: 22RV1-NC: parental control 22RV1 cells; 22S: 22RV1 cells overexpressing SRRM4 (NEPC model); 22SNC: vector control for 22S cells; 22S8DNC: 22S cells transduced with negative-control lentivirus; 22S8DOE: 22S cells overexpressing LRRC8D; 22S8DKD: 22S cells with LRRC8D knockdown. All experiments were repeated three times. “*” represented *p* < 0.05.

Because NE-like PCa states are characterized by dense-core neurosecretory granules in addition to marker expression [32], ultrastructural features were examined following modulation of LRRC8D. TEM revealed increased dense-core granules in NE-like cells compared with adenocarcinoma controls (*p* < 0.01; Figure 4A,B). LRRC8D knockdown further increased granule abundance (*p* < 0.05), whereas LRRC8D overexpression reduced granule numbers (*p* < 0.01; Figure 4C,D). Consistently, SYP expression was elevated upon LRRC8D knockdown in 22RV1 cells (Figure 4E–G), but was suppressed in LRRC8D-overexpressing PC3 and 22RV1-SRRM4 cells (Figure 4H–K), supporting an inverse relationship between LRRC8D expression and neurosecretory marker levels.

To assess its impact on tumor growth, xenografts derived from LRRC8D-overexpressing NE-like cells (22S8DOE) exhibited significantly reduced tumor growth and smaller tumor volumes compared with vector controls (22S8DNC) (*p* < 0.01; Figure 4L–P). IHC analysis further demonstrated decreased SYP and increased LRRC8D expression in LRRC8D-overexpressing tumors (Figure 4Q,R). These results indicate that LRRC8D limits neurosecretory remodeling and tumor growth in NE-like PCa models.

To investigate the channel function of LRRC8D, the channel activity of VRAC was detected on genetically modified cells, including 22S8DNC, 22S8DKD (22S cells with LRRC8D knockdown), and 22S8DOE cells (22S cells overexpressing LRRC8D) using the MQAE probe. As shown in Figure 4S,T, the relative MQAE fluorescent intensity of 22S8DOE cells was significantly higher than that of 22S8DNC cells, indicating that LRRC8D overexpression enhances swelling-induced anion efflux.

### 3.5. LRRC8D Enhances Cisplatin Responsiveness Independent of AR-Targeted Resistance

**Figure 5:**
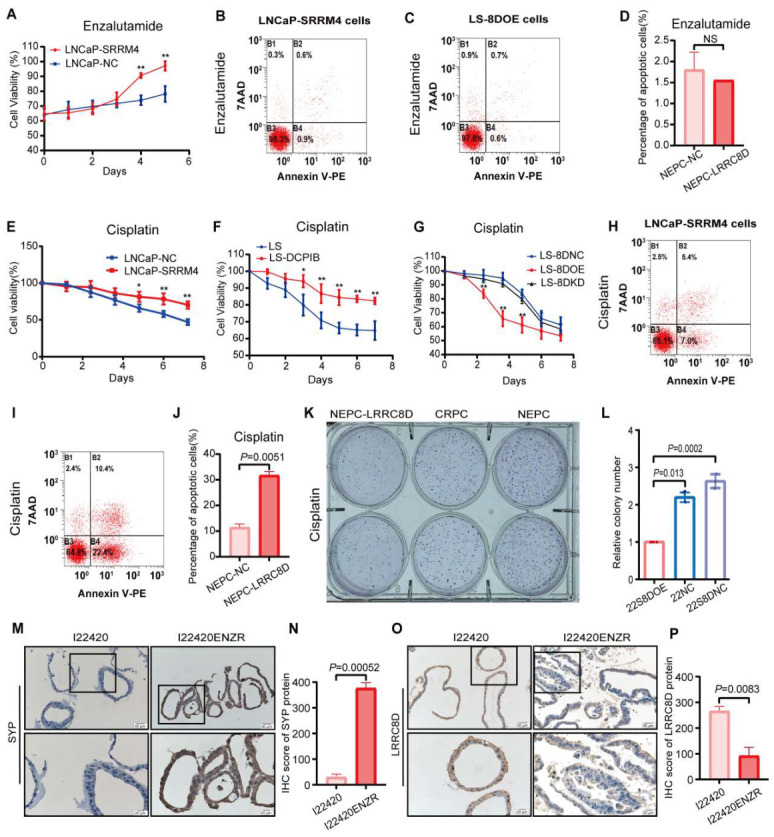
LRRC8D enhances cisplatin sensitivity and is downregulated in enzalutamide (ENZ)-resistant patient-derived organoid (PDO) models. (**A**). Cell viability of LNCaP and LNCaP-SRRM4 cells following treatment with 10 μM ENZ. (**B**). Flow cytometric analysis of apoptotic LNCaP-SRRM4 control cells (NEPC-NC) treated with ENZ. (**C**). Flow cytometric analysis of apoptotic LRRC8D-overexpressing LNCaP-SRRM4 cells (NEPC-LRRC8D) treated with ENZ. (**D**). Quantification of apoptotic cells (%) in the indicated groups. (**E**). Cell viability of LNCaP and LNCaP-SRRM4 cells following treatment with 3 μM cisplatin. (**F**). Cell viability of LNCaP-SRRM4 (LS) cells and DCPIB (5 μM)-pretreated LS cells (LS-DCPIB) following cisplatin treatment. (**G**). Cell viability of LRRC8D-knockdown (LS-8DKD), LRRC8D-overexpressing (LS-8DOE), and control LNCaP-SRRM4 (LS-8DNC) cells after cisplatin exposure. (**H**). Flow cytometric analysis of apoptotic LNCaP-SRRM4 control cells (NEPC-NC) treated with cisplatin. (**I**). Flow cytometric analysis of apoptotic LRRC8D-overexpressing LNCaP-SRRM4 cells (NEPC-LRRC8D) treated with cisplatin. (**J**). Quantification of apoptotic cells (%) in the indicated groups. (**K**). Colony formation of 22RV1-NC (CRPC model), 22RV1-SRRM4 (NEPC model), and LRRC8D-overexpressing 22RV1-SRRM4 cells (NEPC-LRRC8D model) following cisplatin treatment. (**L**). Relative colony number in the indicated groups. (**M**). Immunohistochemical (IHC) staining of SYP in control PDO (I22420) and ENZ-resistant PDO (I22420ENZR). (**N**). IHC score of SYP protein expression. (**O**). IHC staining of LRRC8D in control PDO (I22420) and ENZ-resistant PDO (I22420ENZR). (**P**). IHC score of LRRC8D protein expression. Scale bar: 20 μm. “*” represented *p* < 0.05, “**” represented *p* < 0.01.

NE-like PCa cells are generally refractory to AR-targeted therapies while remaining responsive to platinum-based agents [15]. Given the reduction in LRRC8D expression and VRAC activity observed in NE-like states, therapeutic responsiveness to ENZ and cisplatin was evaluated following modulation of LRRC8D expression. Treatment with ENZ resulted in limited growth inhibition in LNCaP-SRRM4 cells compared with parental LNCaP cells (Figure 5A). Flow cytometric analysis demonstrated no significant difference in ENZ-induced apoptosis between LNCaP-SRRM4 control cells and LRRC8D-overexpressing LNCaP-SRRM4 cells (Figure 5B–D), indicating that LRRC8D overexpression does not alter ENZ responsiveness in this NE-like model.

In contrast, cisplatin response was markedly affected by LRRC8D status. LNCaP-SRRM4 cells displayed reduced sensitivity to cisplatin relative to parental controls (Figure 5E). Pharmacological inhibition of VRAC with DCPIB further attenuated cisplatin-induced growth suppression (Figure 5F), supporting a role for chloride channel activity in platinum responsiveness. Upon direct modulation of LRRC8D, overexpression significantly enhanced cisplatin-induced cytotoxicity, whereas knockdown modestly reduced responsiveness compared with vector controls (Figure 5G). Apoptosis assays further demonstrated that LRRC8D overexpression increased cisplatin-induced apoptotic fractions in SRRM4-induced NE-like cells (Figure 5H–J). Consistently, colony formation assays showed that LRRC8D-overexpressing 22RV1-SRRM4 cells (NEPC model) formed fewer colonies under cisplatin exposure compared with control groups (Figure 5K,L), indicating enhanced suppression of clonogenic survival.

To assess clinical relevance, LRRC8D expression was examined in patient-derived tumor organoids. ENZ-resistant organoids (I22420ENZR) exhibited significantly increased SYP expression and reduced LRRC8D levels compared with parental controls (Figure 5M–P), demonstrating an inverse relationship between LRRC8D expression and NE-associated features in therapy-resistant samples. Collectively, these data indicate that LRRC8D enhances cisplatin sensitivity, likely through mechanisms linked to VRAC function, while exerting minimal effect on AR-targeted therapeutic response, consistent with the phenotypic observations of increased swelling-induced anion efflux.

### 3.6. LRRC8D Is Transcriptionally Regulated by REST and Associated with CAV-1

**Figure 6:**
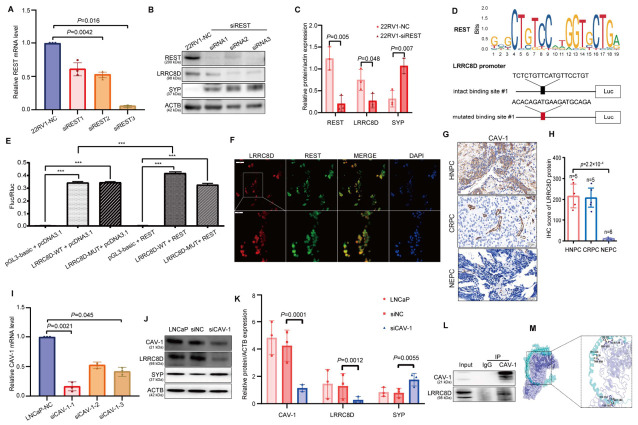
LRRC8D is transcriptionally activated by REST and associates with CAV-1. (**A**). Relative REST mRNA level after REST knockdown using three independent REST-targeting siRNAs. (**B**). Representative immunoblot of REST, LRRC8D, SYP, and β-ACTIN in 22RV1 cells after REST knockdown. (**C**). Quantification of REST, LRRC8D, and SYP protein expression in the indicated groups (n = 3). (**D**). Predicted REST-binding motif from the JASPAR database and schematic of wild-type and mutant LRRC8D promoter constructs used for luciferase reporter assays. (**E**). Dual-luciferase reporter assay assessing the effect of REST overexpression on LRRC8D promoter activity in HEK293T cells using wild-type or mutant promoter constructs. (**F**). Immunofluorescence staining of LRRC8D, REST, and DAPI in LNCaP cells. (**G**). Immunohistochemical (IHC) staining of CAV-1 in tissues from patients with hormone-naive prostate cancer (HNPC), castration-resistant prostate cancer (CRPC), and NEPC. Scale bar: 20 μm. (**H**). IHC scores of CAV-1 in the indicated groups. (**I**). Relative CAV-1 mRNA level after CAV-1 knockdown using three independent CAV-1-targeting siRNAs. (**J**). Representative immunoblot of CAV-1, LRRC8D, SYP, and β-ACTIN in LNCaP cells after CAV-1 knockdown. (**K**). Quantification of CAV-1 and LRRC8D protein expression in the indicated groups (n = 3). (**L**). Co-immunoprecipitation (co-IP) showing the interaction between CAV-1 and LRRC8D in 22S-8DOE cell lysates. (**M**). Structural modeling of the predicted LRRC8D-CAV-1 interaction based on molecular docking analysis.”***” represented *p* < 0.001.

To identify upstream regulators of LRRC8D transcription, the neuronal repressor REST was examined, as REST is frequently reduced in NE-like PCa states [33,34]. REST silencing in 22RV1 cells significantly reduced LRRC8D mRNA and protein levels while increasing SYP protein expression (Figure 6A–C). Bioinformatic prediction identified a REST-binding site in the LRRC8D promoter (Figure 6D), and dual-luciferase assays demonstrated that REST directly enhanced LRRC8D promoter activity (Figure 6E). Immunofluorescence staining showed nuclear localization of REST and predominant membrane and cytoplasmic distribution of LRRC8D (Figure 6F). These findings indicate that REST positively regulates LRRC8D expression.

Given the membrane localization of LRRC8D, potential interaction partners involved in membrane signaling were explored. CAV-1, a membrane scaffolding protein implicated in PCa progression and phenotypic plasticity, was examined [35]. IHC staining demonstrated reduced CAV-1 expression in NE-like PCa tissues compared with adenocarcinoma samples (Figure 6G,H), consistent with the reduction observed for LRRC8D (Figure 2L). Silencing of CAV-1 in LNCaP cells significantly reduced LRRC8D protein levels while increasing SYP protein expression (Figure 6I–K), suggesting a functional association between these proteins. Co-IP analysis showed that LRRC8D and CAV-1 are present in the same protein complex (Figure 6L), indicating their co-existence within a common signaling environment. Molecular docking predicted potential contact residues between LRRC8D and CAV-1, including hydrogen bonds between LRRC8D THR760 and CAV-1 GLU161, THR490-HIS126, GLN529-LYS57, SER533-LYS57, along with an electrostatic interaction between HIS758 and GLU161 (Table 1, Figure 6M). These computational predictions, together with the Co-IP data, raise the possibility of a direct or indirect association, but definitive evidence of direct physical binding requires further validation. Collectively, these findings indicate that REST promotes LRRC8D transcription and that LRRC8D functions in conjunction with CAV-1, forming a membrane-associated regulatory axis that may contribute to altered phenotypes and platinum responsiveness in NE-associated PCa states.

### 3.7. LRRC8D Attenuates STAT3 Signaling and Enhances Cisplatin-Induced Apoptosis

**Figure 7:**
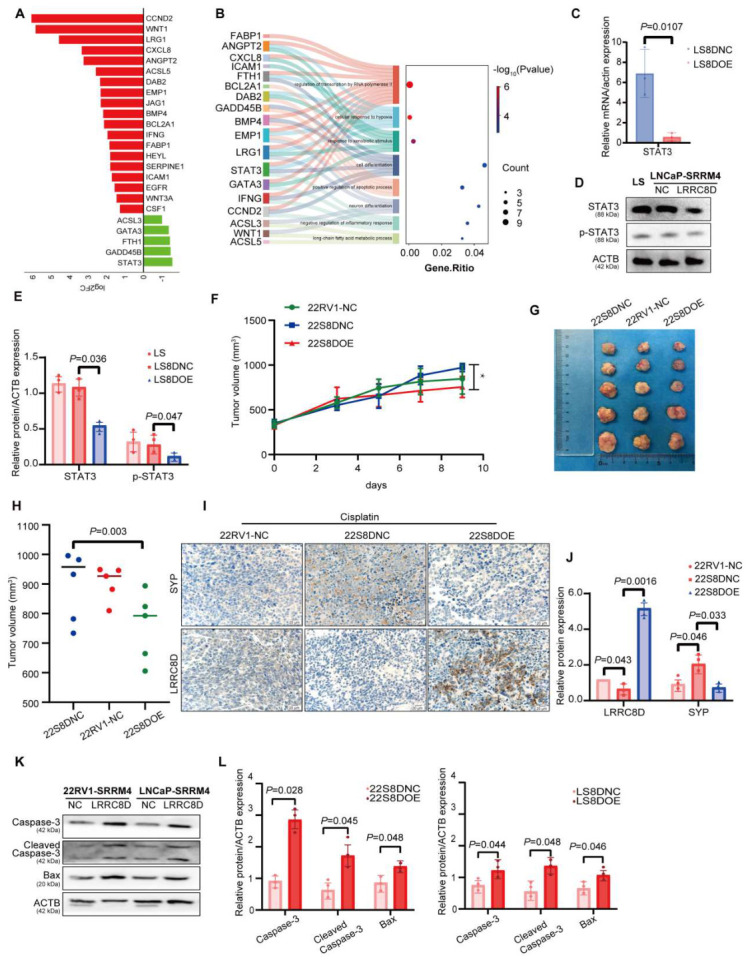
LRRC8D attenuates STAT3 signaling and enhances cisplatin-induced tumor suppression. (**A**). Bar plot showing differentially expressed genes (DEGs) in LS8DOE cells compared with LS8DNC cells. (**B**). Gene ontology (GO) enrichment analysis (biological process category) of DEGs between 22S8DOE and 22S cells. (**C**). STAT3 mRNA expression levels in LS8DOE and LS8DNC cells. (**D**). Representative immunoblot of total and phosphorylated STAT3 in LNCaP-SRRM4 cells with or without LRRC8D overexpression. (**E**). Quantification of total and phosphorylated STAT3 protein levels in the indicated groups (n = 3). (**F**). Tumor growth curves of 22RV1-NC, 22S8DNC, and 22S8DOE xenografts after cisplatin treatment (n = 5). (**G**). Representative images of excised xenograft tumors (n = 5). (**H**). Quantification of tumor volumes after excision (n = 5). (**I**). Immunohistochemical staining and quantification of LRRC8D and SYP expression in xenograft tissues shown in panel G. Scale bar: 20 μm. (**J**). Relative protein expression of LRRC8D and SYP in the indicated groups. (**K**). Representative immunoblot of caspase-3, cleaved caspase-3, and Bax in 22RV1-SRRM4 and LNCaP-SRRM4 cells with or without LRRC8D overexpression following cisplatin treatment (3 μM). (**L**). Quantification of caspase-3, cleaved caspase-3, and Bax protein levels in the indicated groups (*n* = 3). Note: LS8DOE: LNCaP-SRRM4 cells overexpressing LRRC8D; LS8DNC: vector control. All experiments were repeated three times.“*” represented *p* < 0.05.

To investigate downstream pathways associated with LRRC8D, transcriptomic analysis of LRRC8D-overexpressing 22RV1-SRRM4 cells was performed. Differential expression profiling revealed suppression of STAT3 and enrichment of pathways related to cellular differentiation and stress response (Figure 7A,B and Figure S2A). Downstream interaction analysis identified potential regulatory networks among differentially expressed proteins (Figure S2B). Consistent with the transcriptomic data, LRRC8D overexpression significantly reduced Stat3 mRNA levels (Figure 7C) and decreased total and phosphorylated STAT3 protein levels (Figure 7D,E), indicating attenuation of STAT3 signaling.

Given the established role of STAT3 in survival signaling and therapy resistance, the impact of LRRC8D on cisplatin response was further examined in vivo. After cisplatin treatment, xenografts derived from LRRC8D-overexpressing cells exhibited significantly reduced tumor growth compared with vector controls (Figure 7F–H). IHC analysis demonstrated increased LRRC8D and reduced SYP expression in LRRC8D-overexpressing tumors (Figure 7I,J), indicating modulation of NE-associated marker expression. Moreover, LRRC8D overexpression enhanced cisplatin-induced activation of caspase-3 and increased Bax expression (Figure 7K,L), supporting augmented apoptotic response under platinum treatment. Collectively, these results suggest that LRRC8D enhances platinum sensitivity and is associated with reduced STAT3 survival signaling.

## 4. Discussion

This study demonstrates that LRRC8D restrains tumor growth and increases platinum sensitivity in aggressive PCa models. LRRC8D overexpression reduced SYP expression, decreased STAT3 phosphorylation, and enhanced cisplatin-induced activation of caspase-3 and Bax. LRRC8D expression was maintained by REST, and LRRC8D was found to co-exist in a protein complex with CAV-1. LRRC8D overexpression was also associated with reduced STAT3 signaling. Together, these findings suggest that LRRC8D enhances platinum responsiveness and may limit STAT3-associated survival signaling in PCa.

VRACs are recognized for their roles in osmotic balance and drug transport, and LRRC8D is essential for cisplatin entry via LRRC8A/D-containing pores [13,36]. The present findings extend this concept by demonstrating that LRRC8D expression enhances platinum sensitivity in PCa models. In addition, LRRC8D overexpression reduced SYP expression and neurosecretory granule abundance, indicating modulation of NE-associated phenotypic features. Unlike other VRAC subunits linked to calcium signaling, immune regulation, or tumor suppression [37,38,39], LRRC8D appears to couple platinum responsiveness with changes in NE marker expression.

Among VRAC family members, LRRC8D was the only subunit consistently downregulated in NEPC, distinguishing it from LRRC8A, which is required for channel assembly but showed variable expression and minimal association with NE marker expression. These findings suggest that VRAC subunits contribute differentially to PCa biology. Loss of LRRC8D after cAMP treatment or SRRM4 overexpression was associated with reduced channel activity, increased SYP expression, enhanced neurosecretory granule formation, and diminished cisplatin sensitivity, supporting a role in coordinating phenotypic features of aggressive disease with platinum responsiveness. However, knockdown of LRRC8D had a minimal effect on VRAC activity, whereas its overexpression enhanced VRAC function (Figure 4S,T), suggesting that LRRC8D acts as a non-essential, modulatory subunit. Rather than directly participating in pore formation per se, LRRC8D primarily influences channel permeability, substrate selectivity, and gating kinetics.

Reduced cisplatin sensitivity following VRAC inhibition is in line with prior reports that LRRC8A/D-containing channels mediate platinum uptake [36]. In the present study, LRRC8D loss was associated with decreased cisplatin responsiveness and increased dense-core granule accumulation, a morphological feature commonly observed in NE-like states [40]. Given that granule size exceeds VRAC pore diameters [41], the increase in neurosecretory structures is unlikely to result from altered drug transport alone. Instead, impaired chloride flux secondary to LRRC8D reduction may affect vesicular homeostasis, which is implicated in vesicle acidification and granule maturation [42]. Thus, LRRC8D deficiency may simultaneously limit platinum entry and alter secretory phenotypes, potentially contributing to the association between chemoresistance and NE-like features in advanced PCa.

Mechanistically, the regulatory relationships involving REST, LRRC8D, CAV-1, and STAT3 provide a potential framework for understanding LRRC8D function in aggressive PCa. REST has been recognized as a gatekeeper of adenocarcinoma identity by repressing neuronal gene programs, and its loss promotes NE transdifferentiation [43,44]. The observation that REST transcriptionally activated LRRC8D suggests that REST safeguards epithelial lineage not only by silencing neuronal effectors such as SYP but also by maintaining ion channel subunits that constrain lineage plasticity. Unlike established mediators such as SRRM4 or BRN2, which act through splicing or transcriptional reprogramming [7,11], LRRC8D appears to operate by modulating membrane-associated signaling processes. Functional analyses showed that CAV-1 acts upstream of LRRC8D (Figure 6J), and LRRC8D overexpression reduced STAT3 activation (Figure 7D). Co-IP confirmed that LRRC8D and CAV-1 co-exist in the same protein complex (Figure 6L), and computational docking predicted potential contact residues (Figure 6M); however, definitive evidence of direct physical binding requires further validation. Together, these findings support a functional axis in which LRRC8D, in conjunction with CAV-1, influences STAT3-dependent survival and differentiation signals [45]. These findings support coordinated regulation linking ion channel composition, scaffold interactions, and STAT3 activity.

Clinically, these findings suggest that LRRC8D may serve as a potential biomarker in aggressive PCa. Reduced LRRC8D levels were associated with increased SYP expression, diminished VRAC activity, and attenuated cisplatin responsiveness in experimental models, indicating that LRRC8D status may reflect tumor vulnerability to platinum-based therapy. Although validation in clinical cohorts is required, assessment of LRRC8D expression could help identify tumors with altered survival signaling and differential chemotherapy sensitivity. From a therapeutic perspective, modulation of VRAC function or restoration of LRRC8D expression may represent exploratory strategies to enhance platinum responsiveness. However, further mechanistic and translational studies are necessary to determine whether targeting this pathway yields a clinically meaningful benefit.

This study identifies LRRC8D as a modulator of platinum sensitivity and NE-associated features in aggressive PCa models, linking VRAC composition with downstream signaling changes. However, several limitations should be noted. First, the contribution of LRRC8D to VRAC currents was not directly examined in LRRC8D-manipulated cells by patch-clamp analysis. Second, although structural modeling predicted specific residue-level interactions between LRRC8D and CAV-1, these interfaces were not experimentally validated by mutagenesis or functional disruption assays. Third, although REST was shown to transcriptionally regulate LRRC8D, the temporal dynamics of REST loss during disease progression require clarification. Fourth, while overexpression of LRRC8D enhanced cisplatin-induced apoptosis, the specific apoptotic pathways mediating this effect were not identified. Lastly, the co-IP demonstrates that LRRC8D and CAV-1 co-exist in a common complex but do not prove direct physical interaction; additional studies are needed to establish direct binding.

## 5. Conclusions

**Figure 8:**
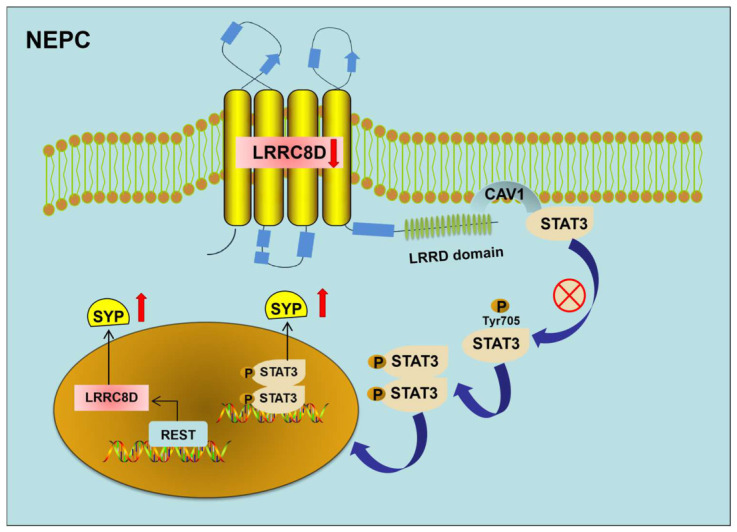
Proposed model of LRRC8D-associated signaling in PCa. The schematic summarizes a regulatory framework involving REST, LRRC8D, CAV-1, and STAT3 in PCa cells. REST maintains LRRC8D expression, whereas stimulation with agents such as cAMP reduces LRRC8D expression and impairs VRAC activity. Reduced LRRC8D is accompanied by altered interaction with caveolin-1 (CAV-1) and increased STAT3 phosphorylation. Activated STAT3 translocates to the nucleus and promotes transcription of NE-associated markers such as synaptophysin (SYP). These signaling changes are associated with NE-like features and reduced platinum sensitivity in aggressive PCa.

Reduced LRRC8D expression characterizes aggressive PCa with neuroendocrine-associated features and diminished cisplatin responsiveness. The model in Figure 8 summarizes REST-dependent maintenance of LRRC8D expression, the co-existence of LRRC8D and CAV-1 in a protein complex, and the association of LRRC8D with reduced STAT3 activation. These observations indicate that ion channel composition may influence tumor phenotype and response to platinum-based therapy in advanced PCa. Modulating LRRC8D expression or function may therefore represent a potential strategy to improve treatment of aggressive disease. Further studies are needed to definitively establish the molecular details of the LRRC8D-CAV-1 relationship and the causal role of STAT3 in mediating the phenotypic effects.

## Figures and Tables

**Table 1:** Functional residue pairs between LRRC8D and CAV-1.

Receptor	Ligand	Hydrogen Bond Interaction	Electrostatic Interaction
LRRC8D	CAV1	THR760-GLU161	HIS758-GLU161
(6M04)	(6SC0)	THR490-HIS126	
		GLN529-LYS57	
		SER533-LYS57	

## Data Availability

All data supporting the findings of this study are included in the article and its Supplementary Materials. Raw datasets generated and analyzed during the current study are available from the corresponding author upon reasonable request.

## Supplementary Materials

The following supporting information can be downloaded at https://www.mdpi.com/article/10.3390/membranes16060198/s1, Figure S1: Validation of NE-like features and VRAC suppression in SRRM4-overexpressing and cAMP-treated PCa cells. (**A**). Swelling-activated chloride currents (I_Cl,swell_) of LNCaP-SRRM4 and 22RV1-SRRM4 cells. (**B**). LRRC8D mRNA expression in PCa datasets curated in Oncomine. (**C**). Relative mRNA expression of SRRM4, SYP, and CHGA in cells with cAMP treatment or SRRM4 overexpression. (**D**). Immunohistochemical staining of SRRM4, SYP, and CHGA in cells with cAMP treatment or SRRM4 overexpression. (**E**). Relative protein expression of SRRM4, SYP, and CHGA in the indicated groups. (**F**). Representative Western blotting showing LRRC8A, LRRC8D, CHGA, SYP, NSE, and β-ACTIN in 22RV1 cells after LRRC8A knockdown. (**G**). Quantification of LRRC8A, LRRC8D, CHGA, SYP, and NSE protein expression in the indicated groups. n = 3. Figure S2: Gene Ontology enrichment and interaction network analysis of differentially expressed proteins in 22S8DOE and 22S8DNC tumor tissues. (**A**). Gene Ontology (GO) enrichment analysis of Biological Process (BP), Cellular Component (CC), and Molecular Function (MF) categories. (**B**). Predicted interaction network of differentially expressed proteins. Table S1: qRT-PCR primer sequences.

## Abbreviations

The following abbreviations are used in this manuscript:

NE	Neuroendocrine
PCa	Prostate cancer
NEPC	Neuroendocrine prostate cancer
VRAC	Volume-regulated anion channels
SYP	Synaptophysin
LRRC8D	Leucine-rich repeat containing 8D
CAV-1	Caveolin-1
STAT3	Signal transducer and activator of transcription
REST	Re1-silencing transcription factor
ADT	Androgen deprivation therapy
CRPC	Castration-resistant prostate cancer
AR	Androgen receptor
ENZ	Enzalutamide
cAMP	Cyclic adenosine monophosphate
Adeno-PCa	Prostatic adenocarcinoma
CDX	Cell-derived xenograft
PCR	Real-time polymerase chain reaction
CHGA	Chromogranin A
NCAM1	Cell-adhesion molecule 1
NSE	Neuron-specific enolase
I_Cl, SWELL_	VRAC swelling-activated anion current
CDS	Coding sequence
SD	Standard deviation
HNPC	hormone-naive prostate cancer
SRRM4	Serine/Arginine Repetitive Matrix 4
