# Supplementary material for: LRRC8D Suppresses Prostate Cancer Growth and Enhances Platinum Sensitivity via Modulation of CAV-1/STAT3 Signaling
Source: Membranes (Basel). 2026 Jun 8;16(6):198. doi: 10.3390/membranes16060198 (PMC13304005; doi:10.3390/membranes16060198)
Supplement: Supplementary file 1 [file membranes-16-00198-s001.zip › membranes-4286826-supplementary.pdf]

Supplementary Materials

Table S1. Primer sequences.

Gene	Sequence（5'→3'）
SRRM4 Forward primer sequence	ACCCTCAGAAAAGCTGGGTC
SRRM4 Reverse Primer Sequence	CGACAGGTCTTGTCTCTCTCC
SYP Forward primer sequence	TTAGTTGGGGACTACTCCTCG
SYP Reverse Primer Sequence	GGCCCTTTGTTATTCTCTCGGTA
CHGA Forward primer sequence	TAAAGGGGATACCGAGGTGATG
CHGA Reverse Primer Sequence	TCGGAGTGTCTCAAAACATTCC
NSE Forward primer sequence	TGAACGTCTGGCTAAATACAACC
NSE Reverse Primer Sequence	GGAAGTTATGTCCGGCAAAG
LRRC8A Forward primer sequence	AGCACTTTGTGTCTATCCTGC
LRRC8A Reverse Primer Sequence	CTGACGGTCGATGACTTTTTGT
LRRC8D Forward primer sequence	ATGACATTCAGCCAACTTACCG
LRRC8D Reverse Primer Sequence	TACTGGCAAACAGACCACCTG
ACTIN Forward primer sequence	CATGTACGTTGCTATCCAGGC
ACTIN Reverse Primer Sequence	CTCCTTAATGTCACGCACGAT

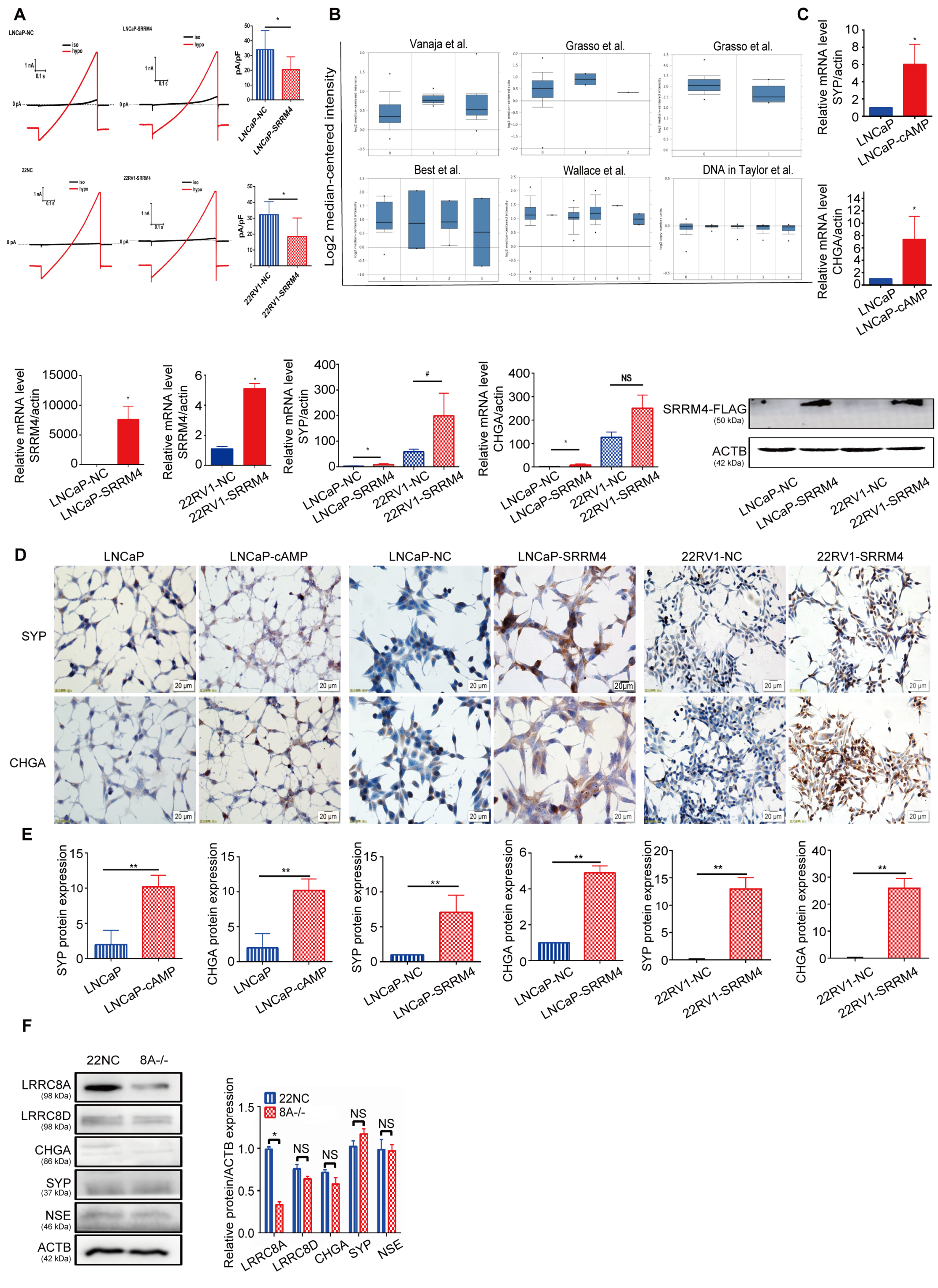

Figure S1. Validation of NE-like features and VRAC suppression in SRRM4-overexpressing and cAMP-treated PCa cells. "*" represented $p < 0.05$, "#" represented $p < 0.05$, "**" represented $p < 0.01$.

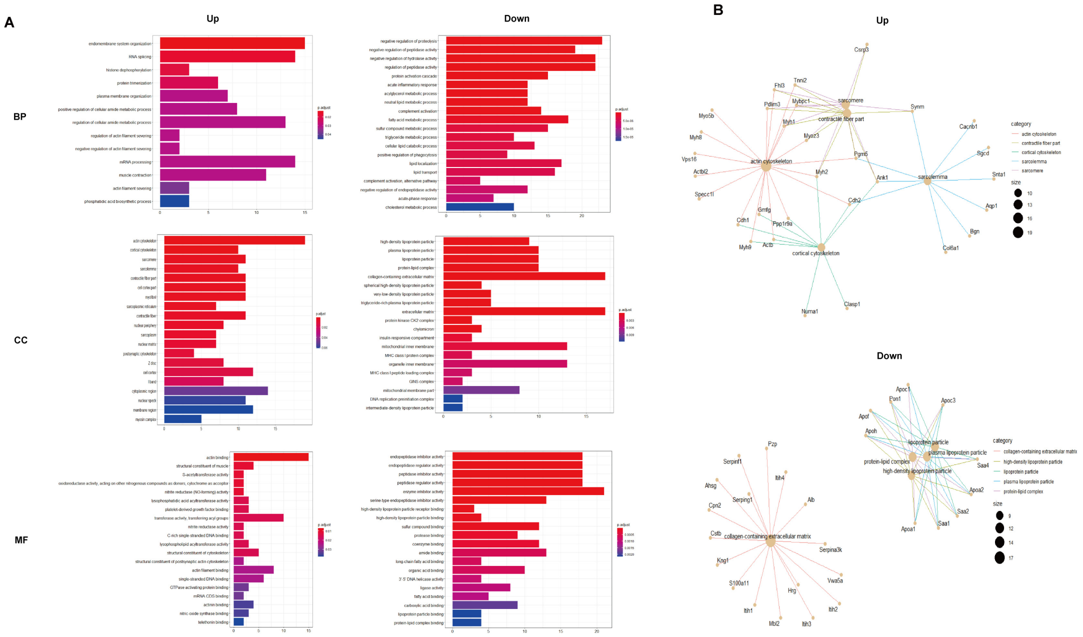

Figure S2. Gene Ontology enrichment and interaction network analysis of differentially expressed proteins in 22S8DOE and 22S8DNC tumor tissues.
